# The Sapap3^−/−^ mouse reconsidered as a comorbid model expressing a spectrum of pathological repetitive behaviours

**DOI:** 10.1038/s41398-023-02323-7

**Published:** 2023-01-30

**Authors:** Hugues Lamothe, Christiane Schreiweis, Lizbeth Sirenia Mondragón-González, Sana Rebbah, Oriana Lavielle, Luc Mallet, Eric Burguière

**Affiliations:** 1 Team “Neurophysiology of Repetitive Behaviours”, Sorbonne Université, Institut du Cerveau – Paris Brain Institute – ICM, Inserm, CNRS, AP-HP, Hôpital de la Pitié Salpêtrière, Paris, France; 2 Université Paris-Est Créteil, Créteil, France; 3 Data Analysis Core, Sorbonne Université, Institut du Cerveau – Paris Brain Institute – ICM, Inserm, CNRS, AP-HP, Hôpital de la Pitié Salpêtrière, Paris, France; 4 Assistance Publique-Hôpitaux de Paris, DMU IMPACT, Département Médical-Universitaire de Psychiatrie et d’Addictologie, Neurosurgery Department, Hôpitaux Universitaires Henri Mondor – Albert Chenevier, Créteil, France; 5 Department of Mental Health and Psychiatry, Global Health Institute, University of Geneva, Geneva, Switzerland

**Keywords:** Neuroscience, Psychiatric disorders

## Abstract

Symptom comorbidity is present amongst neuropsychiatric disorders with repetitive behaviours, complicating clinical diagnosis and impeding appropriate treatments. This is of particular importance for obsessive-compulsive disorder (OCD) and Tourette syndrome. Here, we meticulously analysed the behaviour of Sapap3 knockout mice, the recent rodent model predominantly used to study compulsive-like behaviours, and found that its behaviour is more complex than originally and persistently described. Indeed, we detected previously unreported elements of distinct pathologically repetitive behaviours, which do not form part of rodent syntactic cephalo-caudal self-grooming. These repetitive behaviours include sudden, rapid body and head/body twitches, resembling tic-like movements. We also observed that another type of repetitive behaviour, aberrant hindpaw scratching, might be responsible for the flagship-like skin lesions of this mouse model. In order to characterise the symptomatological nature of observed repetitive behaviours, we pharmacologically challenged these phenotypes by systemic aripiprazole administration, a first-line treatment for tic-like symptoms in Tourette syndrome and trichotillomania. A single treatment of aripiprazole significantly reduced the number of head/body twitches, scratching, and single-phase grooming, but not syntactic grooming events. These observations are in line with the high comorbidity of tic- and compulsive-like symptoms in Tourette, OCD and trichotillomania patients.

## Introduction

Many neuropsychiatric disorders are characterised by pathological repetitive behaviours (RB) such as compulsions, tics, stereotypies, or mannerisms. The exact nature of pathological RB is not always trivial to distinguish and comorbidities impede correct diagnosis and appropriate subsequent treatment [1–3]. This applies especially to two neuropsychiatric disorders with high comorbidity [1, 3, 4]: Tourette Syndrome (TS), a childhood-onset neurodevelopmental disorder characterised by tics, and obsessive-compulsive disorder (OCD), a heterogeneous disorder, of which the most typical form is characterised by obsessions and obsession-dependent compulsions [5]. Tics are defined as sudden, rapid, recurrent, non-rhythmic, stereotyped motor events or vocalisations [5]. Compulsions are clinically described as RBs that individuals feel driven to perform in response to an obsession or according to rules that must be rigidly applied. Although compulsions occur less suddenly than tics, it is not always trivial to correctly distinguish between these two RBs and hence, they could be easily confounded in clinical practice [3, 6]. Furthermore, a third class of disorders with RBs, trichotillomania (TTM), raises yet another important clinical concern. Although TTM is usually easily diagnosed through abnormal RBs such as hair-pulling or skin-picking, it remains debated amongst experts whether these symptoms are of a tic-like or a compulsive-like nature [7].

Rodent self-grooming is recognised as a relevant behavioural output for mapping and probing neural circuits underlying the generation of repetitive behaviours in translational psychiatric approaches [8, 9]. Over the last decade, mice lacking the postsynaptic protein SAP90/PSD95-associated protein 3 (Sapap3^−/−^), which is strongly expressed in the striatum, have been used as the main reference mouse model for compulsive-like behaviours since their phenotype matches with human OCD symptomatology in many ways. In both OCD patients and Sapap3^−/−^ mice, neurophysiological and behavioural components are similarly affected: cortico-striatal transmission is dysregulated [10–15], striatal structure is altered and its activity increased [16–19], OCD-like relevant behaviour such as excessive self-grooming is aberrantly overexpressed despite deleterious consequences, cognitive parameters such as behavioural flexibility are altered [20–22] and anxiety measures are increased [13]. Pharmacotherapy via selective serotonin reuptake inhibitors, which are applied as first-line therapy in OCD, or targeted deep brain stimulation, which is applied in severe, treatment-resistant OCD cases, decreases compulsive-like behaviours in both OCD patients as well as the Sapap3^−/−^ mouse model [13, 23–25]. A neurobiological core candidate in human OCD symptomatology is aberrant orbitofrontal cortex (OFC) neuroanatomy and/or activity [15, 17, 26]. Several studies in Sapap3^−/−^ mice have corroborated the potential implication of the OFC [16, 27]; more specifically the lateral OFC input onto striatal medium spiny neurons (MSN) in Sapap3^−/−^ mice was reduced [28] and their optogenetic excitation restored adapted grooming behaviour and aberrantly elevated striatal firing rates [16].

Dysfunctions of cortico-striatal circuits are consistently reported with the appearance of pathological RBs. These circuits are topographically organised in parallel limbic, associative and sensorimotor loops coursing through the ventral (VS), dorsomedial (DMS) and dorsolateral striatum (DLS), respectively [29–32]. They are known to dynamically interact across these topographically organised loops and are recruited to different extents during learning and automatisation of behaviours [33–37]. While earlier evidence suggest a specific neuropathological connection between compulsive-like behaviours and the so-called ‘associative’ cortico-striatal loop comprising associative cortical regions such as OFC and the dorsomedial and central striatum, other findings suggest the implication of other, complementary dorsal striatal circuits in the generation of pathological RBs in OCD and other comorbid disorders. In the framework of these dynamically interacting cortico-striatal circuits, OCD has recently been discussed as resulting from an imbalance across associative and sensorimotor CSCs [38]. This hypothesis is corroborated by studies demonstrating the implication of the ‘sensorimotor’ cortico-striatal circuits, comprising motor cortices and the dorsolateral striatum, in the generation of pathological RBs [28, 39, 40]. Notably, in the same Sapap3^−/−^ model, which has become the main reference mouse model for studying compulsive-like behaviours in rodents, a recent study revealed that synaptic input from the premotor cortex (M2), as observed in vitro through slice neurophysiological recording, was strengthened in Sapap3^−/−^ mutants, suggesting thus a potential implication of sensorimotor circuits [28]. Yet other studies, including some in patients, point to an implication of the entire dorsal striatal circuits in the generation of several types of pathological RBs [41–48]. The hypothesis of generally compromised cortico-dorsostriatal circuitry mediating RBs is also in line with the observed strong comorbidity of tic- and compulsive-like symptoms in patients with Tourette syndrome or OCD [3, 49–52] and this comorbidity is decisive for successful treatment [49, 50, 53].

Thus, reconsidering cortico-striatal circuitry as a substrate for pathological RBs and taking into account specific indications of an implication of the sensorimotor cortico-striatal loops also in the Sapap3^−/−^ mice [28, 40], we here raised the question whether compulsive-like self-grooming in Sapap3^−/−^ mice might be complemented by RBs of different nature. We first performed a detailed multi-angle video screening to seek for distinct types of pathological RB other than compulsive-like self-grooming. We next confirmed predictive validity by pharmacological treatment of the spectrum of observed pathological RBs. These findings are of crucial interest to redefine the Sapap3^−/−^ mouse model as a model of distinct types of RBs in the light of cortico-dorsostriatal circuitry implication. Indeed, these new results are in line with neurophysiological modifications due to whole-striatal Sapap3 expression patterns, the clinical comorbidity observed in patients, and recent work reconsidering the circuitry affected in this mouse model.

## Materials and methods

### Animals

All experimental procedures followed national and European guidelines and have been approved by the institutional review boards (French Ministry of Higher Education, Research and Innovation; APAFiS Protocol no. 1418-2015120217347265). Animals were group-housed in ventilated standard cages in groups of up to six animals per cage; they were maintained in a 12-h light/dark cycle (lights on/off at 8:00 am/8:00 pm, respectively), and had ab libitum food and water access. Sapap3^−/−^ mutant mice and Sapap3^+/+^ littermates (wt) were generated in heterozygous breeding trios of C57BL/6J background in the animal facility of the Paris Brain Institute. Founders for the Sapap3^−/−^ colony were kindly provided by Dr G. Feng, MIT, Cambridge, USA.

A total of 92 animals (hereof *n* = 16 females and *n* = 76 males) were used in this study and systematically genotyped for the presence or absence of the Sapap3 protein during weaning period following previously described procedures [13]. In detail, *n* = 55 Sapap3^−/−^ mice were used for lesion evaluation (hereof, *n* = 17 were used to evaluate the effect of hindpaw nail clipping after 2 days and *n* = 16 to evaluate this effect after 2 weeks); *n* = 9 of each Sapap3^−/−^ and wildtype mice for screening of repetitive behaviours; and *n* = 15 Sapap3^−/−^ mice for aripiprazole treatment. Sample sizes were chosen according to previous publications using a comparable number of animals for evaluating repetitive behaviours (e.g., see [54] *n* = 10 Sapap3^−/−^ mice; *n* = 12 Sapap3^−/−^ mice [55]) as well as according to pharmacological treatment in the same mouse model (*n* = 9–11 Sapap3^−/−^ mice [13]). Non-parametric permutation tests were conducted as a robust statistical approach based on resampling in order to increase confidence in the obtained results.

Animals for naive behaviour were chosen randomly from the available colony pool of Sapap3^−/−^ adult mice (>4 months of age) and age-matched wildtype littermates. For the aripiprazole experiment, adult animals were briefly (1–2 min) observed in their homecages for signs of increased grooming activity, for signs of anxiety (e.g., eye squinting, anxious crouching, freezing, hiding away from the experimenter) and general quality of fur and skin. The animals were selected in a range between mild to moderate phenotypes. For the nail clipping experiments, we selected adult Sapap3^−/−^ animals showing a range of mild to severe skin lesions of different shapes and locations.

### Video acquisition

**Fig. 1:**
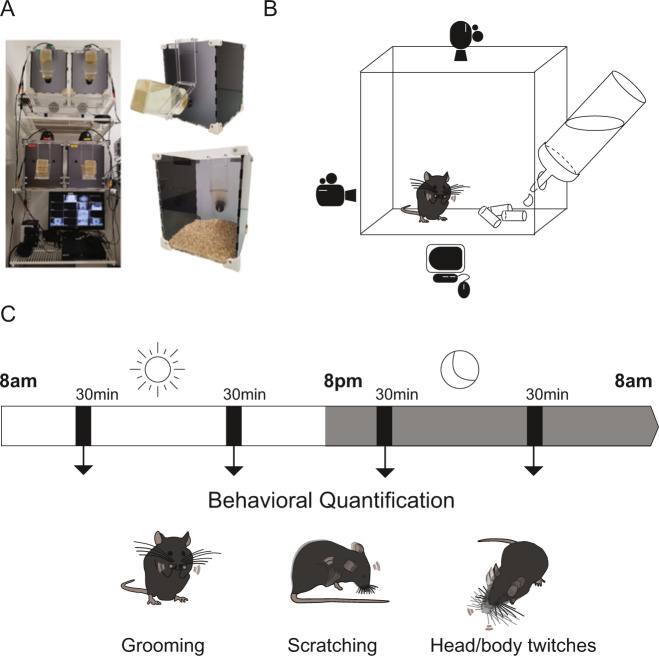
Behavioural assessment of Sapap3^−/−^ mice. **A** Photographs of custom-made apparatus for behavioural assessment, consisting of four acrylic chambers, each equipped with top and side cameras, connected to a digital video-recording system. **B** Detailed graphic illustration of a single video chamber with ad libitum water and food access. **C** Time scale of behavioural assessment. Mice were video-recorded in the behavioural apparatus for 24 h. Four intermittent time bins of 30 min each (i.e., a total of 2 h) were manually analysed offline for repetitive behaviours including self-grooming, head-body twitches and hindpaw scratching. The scored time bins were distributed regularly across the light/dark circadian following previous protocols (Welch et al., 2007).

For the detailed behavioural characterisation in naive mice, animals were temporarily separated from their littermates for a continuous video-recording session of 24 h in video-recording apparatuses. An innovative recording setup has been custom-made for the purpose of our experiment to allow for detailed behavioural analysis. The setup was equipped with four behavioural boxes (black acrylic side walls, opaque front wall, transparent back wall; 20 cm (l) × 20 cm (w) × 25 cm (h)). Each box was equipped with a side and a top camera (25 fps) and connected to a digital video-recording system (KKMoon, Shenzhen Tomtop Technology) (Fig. 1A, B). The boxes were filled with standard wood bedding; ad libitum water and food were provided. As in the animals’ regular housing conditions, light was on between 8 am and 8 pm and infrared illumination was on between 8 pm and 8 am. In addition, a commercially available system with similar specificities (StereoScan, CleverSys®, Reston, VA, USA) has been used to complement our video-recording boxes.

### Pharmacological treatments with aripiprazole

Sapap3^−/−^ mice (*n* = 15) were weighted and placed inside the video acquisition system at 10 am. Animals were habituated to the environment for 30 h prior to injections as well as to handling and restraining procedures. At 4 pm the following day, half the animals were injected first with vehicle solution (0.9% sterile solution with 1% Tween 80 and 1% sterile DMSO; 0.1 ml/10 g) and 24 h later with aripiprazole (1.5 mg/kg in vehicle solution, 0.1 ml/10 g) [56, 57]. The other half of the animals received first an aripiprazole injection, followed by a vehicle solution injection a week later to allow for a sufficient washout period of aripiprazole. In that condition, animals were taken out of the video-recording apparatus 24 h after aripiprazole treatment and re-habituated 1 week later to handling, restraining and to the apparatus for 30 h prior to vehicle injections.

### Video analysis

For behavioural assessment, videos were manually analysed offline using a freely available scoring software (Kinovea, 0.8.15, www.kinovea.org), which allows us to tag each individual scored event and to export timestamps of tagged behaviours [16]. The experimenter scoring the behaviour was blind to genotype and treatment and the order of the scored videos was randomised.

For detailed behaviour characterisation in naive mice, four time segments of 30 min were defined across 24 h: 10–10 h30 am, 6–6:30 pm, 9–9 h30 pm, and 4–4 h30 am (Fig. 1C). This selection of time segments comprised dark/light cycle episodes as previous studies including those using automated assessment of grooming [54] and was primarily based on the first study reporting the excessive grooming phenotype in the Sapap3^−/−^ mice [13].

For the behavioural assessment under aripiprazole, one time segment per mouse was selected for video analyses according to the pharmacokinetics of the compound and following procedures of previously published assays [56, 57]. The segment started at 9 pm and lasted until reaching 30 min of active behaviour. Concretely, an independent person randomised the order of videos for mice and treatment (vehicle or aripiprazole) and relabelled the videos in a pseudorandom manner. The expert scorer was blind to genotype and treatment during the entire scoring process.

The proportion of sleep episodes, interspersed during 30 min of active behaviour, was additionally quantified both in the behavioural assessment of naive wildtype and Sapap3^−/−^ mice as well as in aripiprazole-treated Sapap3^−/−^ mice.

### Motion estimation using DeepLabCut

To quantify animal motion during vehicle and aripiprazole treatment, we used an open-source Python package for body part tracking: DeepLabCut (version 2.2.1) [58, 59], with CUDA Toolkit (11.2) and Tensorflow (2.8.0). We used the DeepLabCut toolbox according to the protocol published in [59]. Briefly, the DeepLabCut toolbox was used to extract frames from selected videos, manually annotate body parts of interest from those frames, form a training dataset to train a convolutional neural network, train the neural network and evaluate the performance of the network. Specifically, we labelled 200 frames per mouse (*n* = 15, Sapap3^−/−^) taken from one video per animal, with all videos corresponding to the hour directly following the video-recording used to assess the vehicle or aripiprazole effect. To capture gait and head-turning while standing still, we targeted the hump on the centre back as an estimate for body centre, and the middle site between the ears as a marker for head location. A total of 90% of the frames were used to form a database of training. We used a ResNet-50-based neural network for 30,000 iterations [60]. We validated with 50.000 number of shuffles and found a test error of 9.07 pixels and a training error of 6.19 pixels (image size was 704 by 576). We then used a *p* cut-off of 0.6. This network was then applied to analyse 15 one-hour videos that we used to assess repetitive behaviours in both the vehicle and aripiprazole conditions.

To estimate the activity and locomotion of the mice during the awake states, the X and Y coordinates of the tracked head and centre back marker, determined with DeepLabCut, were imported into Python (v.3.8.10) and processed with custom scripts. The instantaneous speed of the head and centre back marker was determined between two frames (25 fps) by deriving the markers’ positions over time. The activity and distance travelled was estimated with the X and Y coordinates of the head and centre back marker by calculating the Euclidian distance between two frames and its cumulative total distance. The pixel-to-cm conversion for each video was determined by taking as a scale reference the distance between the head and centre back markers.

### Ethogram

#### Self-grooming

Self-grooming behaviour is defined as a rostro-caudal sequence of four typical, distinct, often intermittently executed phases as previously described in the literature for rodent syntactic grooming [61, 62]. In our study, we distinguished two different types of grooming bouts. Short grooming bouts (<3 s) are predominantly composed of only one of the four grooming phases (Supplementary Fig. 1 and Supplementary Video 1), and long grooming bouts (>3 s) are composed of multiple self-grooming phases separated by less than 1 s from each other.

#### Head/body twitches

Head/body twitches were defined as rapid, sudden repetitive behaviours, consisting of a single movement and corresponding to axial jerks as described in mouse models of tic-like behaviours [63, 64] (Supplementary Videos 1 and 2).

#### Scratching behaviour

We defined scratching behaviour as a rhythmic movement of the hind limbs interacting with more rostral parts of the body [65]. The targeted body parts varied between individuals in snout, area around the eyes, upper forehead, neck, between shoulders and on the back (Supplementary Videos 1 and 3).

### Nail clipping assay

We selected 20 mice (13 male and 7 female) Sapap3^−/−^ mice with lesions of different severity grades to perform hindpaw nail clipping under isoflurane anaesthesia (Isovet, Centravet, 1000 mg/g). Using small surgery scissors, we removed the pointy part of the hindpaw claws without hurting the nailbed. Clipped nails were disinfected with 10% betadine solution (Vétédine, Vétoquinol) and mice were placed back into their homecages with their littermates. Lesions were scored at three different time points: before nail clipping procedure, 2 days and 2 weeks after nail clipping. Hereby, a common pool of *n* = 13 mice was assessed on all three time points; *n* = 4 additional mice were assessed only prior to and 2 days after nail clipping; *n* = 1 mouse was additionally assessed only prior to and 2 weeks after nail clipping treatment. Lesion scores were determined according to the following definitions: absence of lesions (score 1); mild fur and skin lesions without blood crusts (score 2); moderate fur and skin lesions with blood crusts (score 3); tissue missing with blood crusts or open, wet skin (severe lesion) (score 4).

### Statistical analysis

For statistical analysis, we used the following non-parametric tests under R version 3.4.0 (https://www.r-project.org/): Spearman tests for assessing correlations, Mann–Whitney *U* testing for between-group comparisons, Wilcoxon signed-rank test for evaluating treatment effects (nail clipping, aripiprazole), and Aligned Rank Transformation Analysis of Variance for testing factor interactions (package ARTool v0.10.6). We additionally calculated Wilcoxon effects sizes for all repetitive behaviours under aripiprazole treatment, and conducted non-parametric, paired or unpaired permutation tests to analyse each response variable of the aripiprazole or naive behavioural dataset, respectively, which did not meet the assumptions of normality and homogeneity of variance. Hereby, the number of iterations was set to 10,000. The level of statistical significance was set at *p* values < 0.05. Permutation tests were conducted using R version 4.1.0 (R Development Core Team, 2021). Briefly, permutation tests are robust statistical approaches based on resampling and thus rely on the empirical and not a theoretical distribution. Thus, they can provide more accurate *p* values and can help control the overall type I error rate Finally, after having verified that the assumptions of normality of distribution and homoscedasticity were fulfilled, we used a linear mixed model (LMM) approach to explain the repetitive behavioural variables by treatment and either sedation or injection order as well as their interactions. To account for individual variability, we implemented subject as weight in the model, and performed Type II Wald *χ*^2^ tests to test the significance of the main effects and interactions. For a comprehensive listing of all conducted statistical analyses and their results, see Supplementary Table 1. For estimating the most reliable separation of single versus syntactic grooming events consisting of distinct grooming phases, we used a receiver operating characteristic (ROC) curve, indicating the optimal true-positive rate (sensitivity) of a finding given the least possible probability of a false positive (1 - specificity). The R packages used for the ROC analysis were pROC (v3.6.3) and epiR (v3.6.1). For graphical illustration, we used the packages ggplot2 (v3.2.0.) and reshape2 (v1.4.3.).

## Results

### Sapap3^−/−^ mice express aberrant head/body twitches

**Fig. 2:**
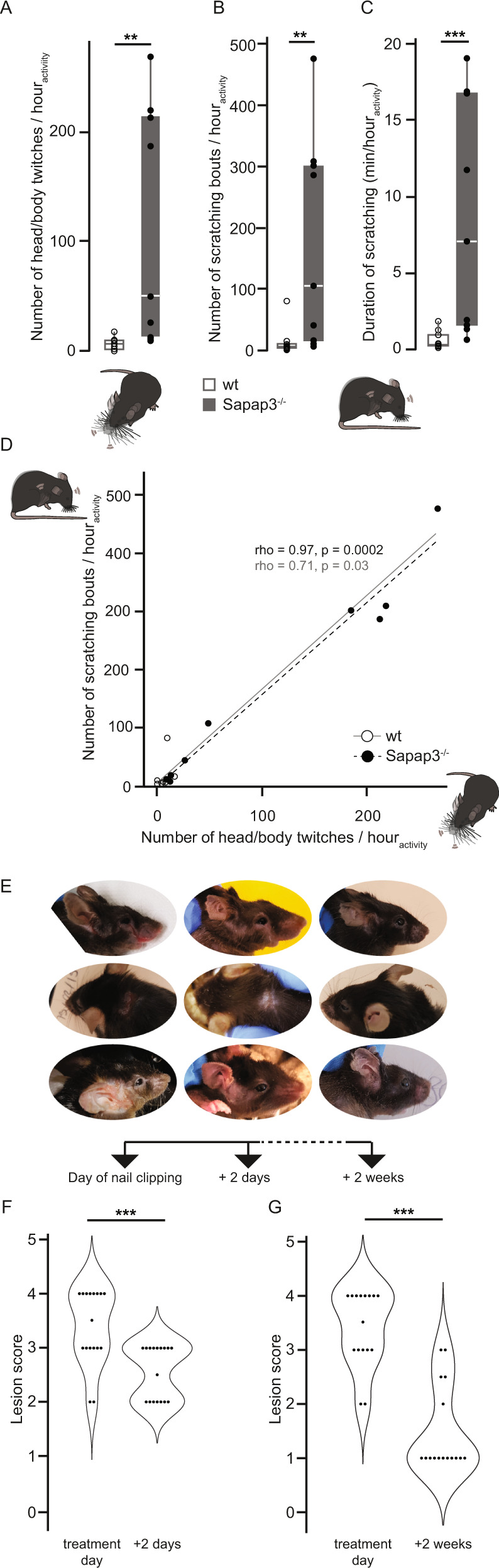
Sapap3^−/−^ mice express aberrant head/body twitches and scratching behaviours. **A** Sapap3^−/−^ mice execute a significant amount of head/body twitches, which are nearly absent in wildtype mice (*n* = 9 mice per genotype; Mann–Whitney *U*, *p* < 0.01). **B** Sapap3^−/−^ mice show a significant amount of hindpaw scratching compared to wildtype control mice (*n* = 9 mice per genotype; Mann–Whitney *U* test, *p* < 0.01). **C** The duration of hindpaw scratching is significantly elevated in Sapap3^−/−^ in comparison to wildtype mice (*n* = 9 mice per genotype; Mann–Whitney *U* test, *p* < 0.001). **D** The number of head/body twitches and scratching bouts correlate positively in both wildtype (Spearman correlation, *p* < 0.05) and Sapap3^−/−^ mice (*n* = 9 mice per genotype; Spearman correlation, *p* < 0.001). **E** Photographs of three individual mice with representative lesions before, and 2 days or 2 weeks after hindpaw nail clipping treatment. **F** Lesions, assessed through a lesion score ranging from no lesions (score = 1) to severe lesions (score = 4), significantly improved already 2 days after clipping the hindpaw claws (*n* = 17 Sapap3^−/−^ mice; Wilcoxon signed-rank test, paired, *p* < 0.001). **G** Lesions are further improved 2 weeks after clipping the hindpaw claws as assessed through a significantly lowered lesion score (*n* = 16 Sapap3^−/−^ mice; Wilcoxon signed-rank test, paired, *p* < 0.001). Box plots illustrate the first and third quartiles; whiskers indicate the minimum and the maximal value of each dataset at no further than 1.5 interquartile range. The indicated average is the median. Quartiles of Sapap3^−/−^ and wildtype mice are plotted in grey or white, and individual data points are in filled black and empty black dots, respectively. **p < 0.01, ***p < 0.001.

Given the clinical reality of tic-like and compulsive-like comorbidity and recent publications reconsidering the purely compulsive-like nature of aberrant self-grooming in the Sapap3^−/−^ mouse [28, 40], we performed a precise screening for other RBs than self-grooming, especially those, which might resemble tic-like movements. Indeed, we detected a very short and sudden type of repetitive behaviour, which is nearly absent in wildtype but significantly present in Sapap3^−/−^ mice (median_wt_ = 6.3 vs. median _Sapap3_^−/−^ = 49.7; Mann–Whitney *U*: *W* = 76, *p* = 0.002; non-parametric permutation test: *p* = 0.01) (Fig. 2A and Supplementary Videos 1 and 2). These repetitive behaviours consist of rapid head/body twitches. This observed sudden, rapid recurrent, non-rhythmic execution of a single movement in the Sapap3^−/−^ model strongly resembles the clinical definition of tics in human patients [5] as well as what has been described for rodent models of tic-like behaviours [57, 63], suggesting face validity of the observed phenotype.

### Typical skin lesions of Sapap3^−/−^ mice are likely provoked by excessive scratching, a repetitive behaviour distinct from syntactic self-grooming

In addition to head/body twitches, we furthermore detected a prominent number of scratching events, which consist of the rapid, repeated beating of the hindpaw against various body parts (such as snout, areas surrounding the eyes and the ears, the neck, between the shoulders etc.), and which have to be distinguished from syntactic grooming, a stereotypically enchained sequence of segregate phases, which is well-conserved in its choreography in all rodents [9, 61, 62]. The amount of scratching events was significantly increased in Sapap3^−/−^ compared to wildtype mice (median_wt_ = 5.7 vs. median_Sapap3_^−/−^ = 106.3; Mann–Whitney *U*: *W* = 73, *p* = 0.003; non-parametric permutation test: *p* = 0.02) (Fig. 2B and Supplementary Videos 1 and 3). The duration of scratching, significantly larger in Sapap3^−/−^ mice, further corroborates the importance of this phenotype (median_wt_ = 0.3 min/h_activity_ vs. median _Sapap3_^−/−^ = 7.1 min/h_activity_; Mann–Whitney *U*: *W* = 76, *p* = 0.0008; non-parametric permutation test: *p* = 0.01) (Fig. 2C). The number of head/body twitches correlated significantly with the number of scratching events (Spearman correlation – wt: *S* = 34.64, rho = 0.71, *p* = 0.03; Sapap3^−/−^: *S* = 4, rho = 0.97, *p* = 0.0002) (Fig. 2D).

During scratching, the hindpaw exerts a strong power onto targeted body areas, including body areas such as the neck or back, which are not touched by the forepaws during the self-grooming sequence. The quality of this event is rather violent and best described as a ‘beating’ of the hindpaw against the body [66]. Given the large frequency and duration of scratching behaviour in Sapap3^−/−^ mice, the occasional detection of blood underneath the hindpaw claws of mice with lesions, the inherent violence of the movement and the observation that a proportion of principal lesions were detected in the neck and/or back of the animals, i.e. body locations, which are not prominently involved in self-grooming behaviour, we established the alternative hypothesis that the flagship-like phenotype of facial and body lesions in Sapap3^−/−^ might be provoked by scratching instead of self-grooming. We therefore screened a large number of Sapap3^−/−^ mutants in the colony (*n* = 55 Sapap3^−/−^ mice) to revisit the most prominent lesion locations on their bodies and found that more than 30% of the lesions were indeed in body locations, which are not touched during the syntactic self-grooming sequence, namely the neck or back (Supplementary Fig. 1A). We analysed a subpopulation of these animals (*n* = 32) more in detail and found that in about 81% of these animals, the principal lesion was accompanied by further lesions at multiple sites including the snout (12.3%), eyes (16.4%), ears (34.2%), top of the head (2.7%), neck (24.7%) or back of the animals (9.6%) (Supplementary Fig. 1B).

Out of the colony pool used to evaluate the lesion locations, we next selected Sapap3^−/−^ mice with representative lesions of various degrees of severity. In these representative individuals, we clipped the sharp tip of exclusively the hind- not forepaw nails without hurting the nailbed. We assessed the severity of the lesions longitudinally, prior to nail clipping, and 2 days or 2 weeks after hindpaw nail clipping. We applied a lesion score determined by the absence of fur, skin or tissue (see Materials and methods section for details). Stark improvement of lesion scores was already clearly detectable in all mice after only 2 days following nail clipping treatment (*n* = 17 mice; Wilcoxon signed-rank test, paired; *V* = 0, *p* = 0.0005) (Fig. 2E, F), and further improved when screened after 2 weeks (*n* = 16; Wilcoxon signed-rank test, paired; *V* = 0, *p* = 0.0002) (Fig. 2E, G).

### Single-phase grooming events are more exaggerated than syntactic grooming in Sapap3^−/−^ mice

**Fig. 3:**
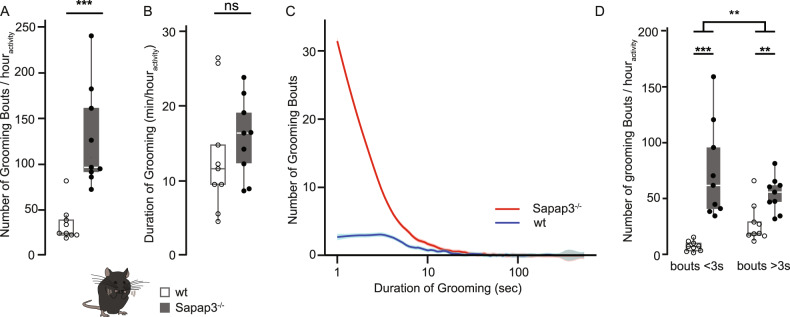
Short, single-phase grooming events are more exaggerated than syntactic grooming in Sapap3^−/−^ mice. **A** Sapap3^−/−^ mice show significantly more grooming events compared to wildtype controls (Mann–Whitney *U* test, *p* < 0.001). **B** Total grooming duration is comparable between Sapap3^−/−^ and wildtype mice (Mann–Whitney *U* test, *p* = ns). **C** Self-grooming behaviour of Sapap3^−/−^ mice compared to wildtype mice is characterised by a large proportion of grooming events of short duration. The *x*-axis is depicted on a log_10_ scale. **D** Both short grooming events (<3 s duration) as well as long grooming events (>3 s duration) were significantly enhanced in Sapap3^−/−^ mice compared to wildtype controls (Mann–Whitney *U*, *p* < 0.001 and *p* < 0.01, respectively). Self-grooming behaviour depended both on genotype and bout length (ART ANOVA, *p*_genotype*grooming type_ < 0.01). All plots illustrate data from *n* = 9 Sapap3^−/−^ and *n* = 9 wildtype mice; box whisker plots were designed as described in the legend of Fig. 2. **p < 0.01; ***p < 0.001; ns non-significant.

Having detected two novel RB phenotypes in the Sapap3^−/−^ mice and having observed that the prominent, typical lesions are inflicted probably by hindpaw scratching, we revisited in detail the self-grooming behaviour in these mice, a highly stereotypical enchainment of four distinct phases [9, 62, 67]. Increased self-grooming in Sapap3^−/−^ mice is usually quantified in the literature either via increased number of grooming events [13, 54] or via increased grooming duration [13, 24, 54]. In our detailed analysis, we decided to pay particular attention to the qualitative grooming heterogeneity observed in mice. We distinguished between both syntactic grooming composed of distinct rostro-caudal phases chained in sequence, and a deviating type consisting of a more sudden isolated short single-phase grooming event. When these two types of grooming were merged together, we observed a significantly increased number of grooming events in Sapap^−/−^ mice (median_wt_ = 24.9 vs. median _Sapap3_^−/−^ = 96.7; Mann–Whitney *U*: *W* = 80, *p* = 0.00008; non-parametric permutation test: *p* = 0.004) (Fig. 3A). However, surprisingly, we did not observe a significant difference in grooming duration between wildtype and mutant mice (median_wt_ = 11.6 min/h_activity_ vs. median _Sapap3_^−/−^ = 16.4 min/h_activity_, Mann–Whitney *U*, *W* = 51, *p* = 0.39; non-parametric permutation test: *p* = 0.4) (Fig. 3B). We first excluded that differences in sleep duration between Sapap3^−/−^ and wildtype mice might be a confounding factor in our grooming dataset (sleep: median_wt_ = 33.2 min vs. median _Sapap3_^−/−^ = 34.7 min; Mann–Whitney *U*: *W* = 41, *p* = 1) (Supplementary Fig. 2A). Thus, we next systematically investigated the distribution and quality of individual grooming events. We indeed detected a difference in the distribution of grooming bout lengths between Sapap3^−/−^ and wildtype controls with a substantial number of grooming events falling into the short event spectrum of the distribution (Fig. 3C). To analyse whether these short grooming events corresponded to short events consisting of a single grooming phases only, we performed a fine-scale scoring analysis, distinguishing individual grooming phases (*n* = 608 number of grooming events in *n* = 4 Sapap3^−/−^ mice; Supplementary Fig. 2B). Applying ROC curve estimations to our full-second binned data, we calculated that short events in our dataset consisting of a single grooming phase and those being composed of distinct grooming phases were best separated by a duration of 3 s (true-positive rate/sensitivity_3s_ = 87.2%; false positive rejection rate/specificity_3s_ = 61.5%; Supplementary Fig. 2C). When classifying all scored grooming events (*n* = 1737 in *n* = 9 mice per genotype) into these two categories, Sapap3^−/−^ mice showed an aberrantly higher number of both short and long grooming bouts (short single-phase grooming bouts: median_Sapap3-/-_ = 61.9; median_wt_ = 7.2, Mann–Whitney *U*: *W* = 81, *p* = 0.0004, non-parametric permutation test: *p* = 0.004; long syntactic grooming bouts: median_Sapap3-/-_ = 56.0; median_wt_ = 18.9, Mann–Whitney *U*: *W* = 71, *p* = 0.006, non-parametric permutation test: *p* = 0.008; Fig. 3D). Although this effect was present in both types of grooming events, the genotype effect depended on the type of grooming (Aligned Ranks Transformation ANOVA (ART ANOVA): *p*_GT*Grooming category_ = 0.01; Fig. 3D). The proportion of short-single-phase to long-syntactic grooming was genotype-dependent: while single-phase grooming events formed about half the number of all grooming events in the Sapap3^−/−^ mice (grooming < 3 s: median_Sapap3-/-_ = 56.3%; median_wt_ = 21.8%, Mann–Whitney *U*: *W* = 79, *p* value = 0.0002), wildtype mice had a significantly higher proportion of long, syntactic grooming events (grooming > 3 s: median_Sapap3-/-_ = 44.7%; median_wt_ = 77.4%; Mann–Whitney *U*: *W* = 2, *p* value = 0.0002; Aligned Ranks Transformation ANOVA (ART ANOVA): *p*_GT*Grooming category_ = 8.7 × 10^−10^; Supplementary Fig. 2D). Lastly, we explored potential confounds between self-grooming and other types of RB such as scratching, which we report here as a novel type of RB. Indeed, when summing up total grooming duration as well as scratching duration, we confirmed that the total duration of RBs in Sapap3^−/−^ was also significantly increased in our dataset (Mann–Whitney *U*: *W* = 65, *p* = 0.003), consistent with previous studies [13]. Taken together, besides the increased number of syntactic self-grooming events previously described, we demonstrated here that exaggerated self-grooming reported in Sapap3^−/−^ mice was prominently due to elevated onsets of the sub-category of short grooming events.

### Excessive head/body twitches, scratching and short grooming events are associated in Sapap3^−/−^ mice

**Fig. 4:**
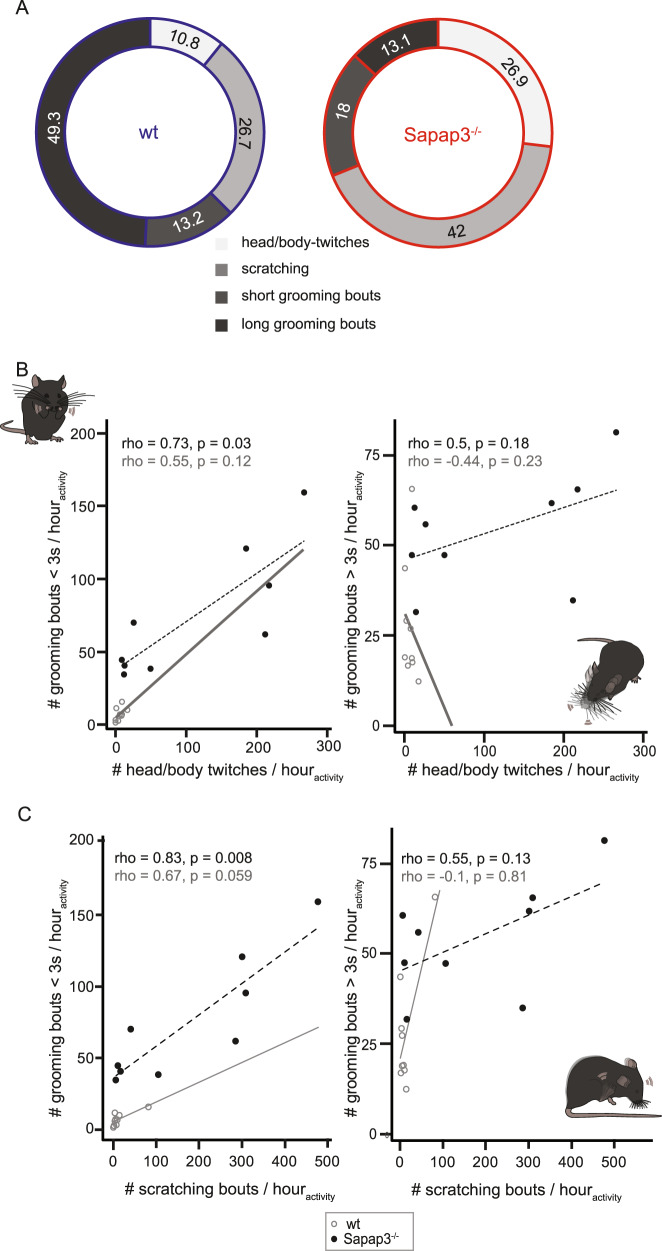
Excessive head/body twitches, scratching and short grooming events are associated in Sapap3^−/−^ mice. **A** The proportion of novel detected repetitive behaviours in Sapap3^−/−^ mice outweighs previously reported syntactic self-grooming behaviour (Pearson’s *χ*^2^ test, *p* < 0.0001). **B** Head/body twitches positively correlate with short, single-phase grooming but not long, syntactic grooming bouts in Sapap3^−/−^ mice (Spearman correlation, *p* < 0.05, *p* = ns, respectively). **C** Scratching bouts also correlate positively with short, single-phase grooming but not long, syntactic grooming bouts in Sapap3^−/−^ mice (Spearman correlation, *p* < 0.01, *p* = ns, respectively). Correlation estimates are plotted in a grey solid line or a dotted black line for wildtype or Sapap3^−/−^ mice (*n* = 9 animals per genotype), respectively.

Next, we analysed the distribution between the four different types of observed RBs, namely head/body twitches, scratching, short and long self-grooming events, as well as the correlations among them. While all four RBs formed part of a normal phenotype in wildtype mice, they were significantly more present in Sapap3^−/−^ mice and their distribution was also significantly different (Pearson’s *χ*^2^ test: *χ*^2^ = 44.1, df = 3, *p* = 1.5 × 10^−9^; Fig. 4A). Head/body twitches positively correlated with short grooming events in Sapap3^−/−^ mice only (Spearman correlation: Sapap3^−/−^: *S* = 32, rho = 0.73, *p* = 0.03; wt: *S* = 53.7, rho = 0.55, *p* = 0.12), but not with long grooming sequences (Spearman correlation: Sapap3^−/−^: *S* = 60, rho = 0.5, *p* = 0.18; wt: *S* = 173, rho = −0.44, *p* = 0.23) (Fig. 4B).

The number of scratching events positively correlated with short but not long grooming events in Sapap3^−/−^ mice (Spearman correlation: short grooming events: *S* = 20, rho = 0.83, *p* = 0.008; long grooming events: *S* = 54, rho = 0.55, *p* = 0.13) (Fig. 4C); no such significant correlation was found in wildtype mice (Spearman correlation: short grooming events: *S* = 40, rho = 0.67, *p* = 0.06; long grooming events: *S* = 132, rho = −0.1, *p* = 0.81) (Fig. 4C). Finally, the number of scratching events and head/body twitches significantly correlated positively in both genotypes (Spearman correlation: Sapap3^−*/*−^: *S* = 4, rho = 0.97, *p* = 0.0002; wt: *S* = 34.6, rho = 0.71, *p* = 0.03) (Fig. 2D).

### Head/body twitches, short grooming bouts and scratching events were selectively reduced by aripiprazole, a first-line pharmacological treatment for Tourette syndrome

**Fig. 5:**
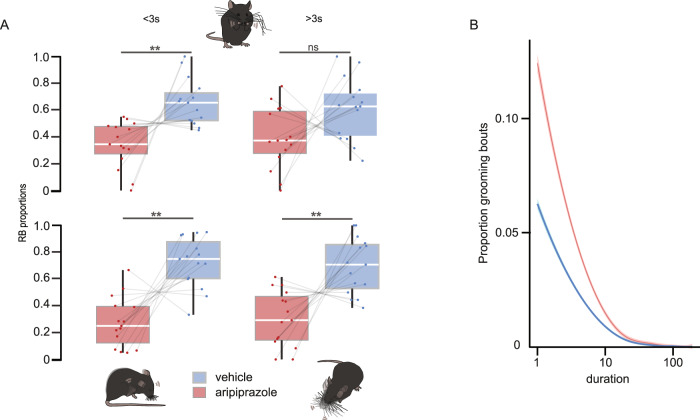
Short grooming bouts, head/body twitches and scratching were reduced by aripiprazole. **A** Acute treatment with aripiprazole (1.5 mg/kg) significantly reduced the number of single-phase grooming, head/body twitches and scratching (Wilcoxon signed-rank test: all *p* < 0.01; non-parametric, paired permutation test: all *p* < 0.01), but not the number of syntactic grooming events (Wilcoxon signed-rank test: *p* = 0.08 and non-parametric, paired permutation test: *p* = 0.09). Plotted are the proportions of number of RB events under aripiprazole treatment and the sum of the number of RB events (vehicle + aripiprazole) of individual mice. **B** Aripiprazole in particular shorter grooming events in Sapap3^−/−^ mice. The *x*-axis is depicted on a log_10_ scale. Box whisker plots were designed as described in the legend of Fig. 2. Vehicle and aripiprazole conditions are colour-coded in blue and red, respectively. *p < 0.05, **p < 0.01, ns non-significant.

Although face validity, i.e., the close phenomenological similarity of tics in human patients and rapid recurrent repetitive behaviours observed in the Sapap3^−/−^ mice, seems to point to a recapitulation of a common aetiology, it is insufficient to draw conclusions about the nature of the observed rodent behaviour. On top, face validity remains the most intuitive but at the same time subjective and prone to anthropomorphic interpretations [68]. Thus, in order to investigate the nature of head-body twitches, scratching, short and long grooming events, and to question if they belong to the same symptomatologic categories, we pharmacologically challenged the predictive validity of these different types of RB observed in Sapap3^−/−^ mice for a potential tic-like nature. Therefore, we applied the first-line pharmacological treatment for tics, aripiprazole [69–73]. Aripiprazole is an atypical antipsychotic medication with a high in vitro affinity for dopamine 2 receptors (D2R) and has a mixed effect as partial agonist and antagonist on type 1A and 2A serotonin receptors, respectively [74, 75]. Aripiprazole has an elimination half-life of approximately 75 h and stable brain-to-serum concentration is achieved after 6 h following acute injection [76]. We applied a dose of 1.5 mg/kg aripiprazole, which previously had been used to successfully reduce what has been reported as tic-like movements in rodent models [56, 57]. We evaluated the effect of acutely administered aripiprazole on the different types of repetitive behaviours observed in the Sapap3^−/−^ mice, comparing the treatment effect to the behavioural baseline of systemic injection of its vehicle solution $\left({\textstyle{{{{{\mathrm{number}}}}\,{{{\mathrm{of}}}}\,{{{\mathrm{RB}}}}\,{{{\mathrm{after}}}}\,{{{\mathrm{aripiprazole}}}}} \over {({{{\mathrm{number}}}}\,{{{\mathrm{of}}}}\,{{{\mathrm{RB}}}}\,{{{\mathrm{after}}}}\,{{{\mathrm{aripiprazole}}}} + {{{\mathrm{number}}}}\,{{{\mathrm{of}}}}\,{{{\mathrm{RB}}}}\,{{{\mathrm{after}}}}\,{{{\mathrm{vehicle}}}})}}}\right)$. Acute aripiprazole treatment significantly lowered the number (Wilcoxon signed-rank test, paired: *V* = 8, *p* = 0.006; non-parametric, paired permutation test: *p*_permutation; short grooming_ = 0.0023) and total duration of short grooming events (Wilcoxon signed-rank test, paired; *V* = 12, *p* = 0.004) (Fig. 5A). This decrease was most visible the shorter the grooming events (Fig. 5B). We additionally found a reduction in the number of head/body twitches (Wilcoxon signed-rank test, paired: *V* = 8, *p* = 0.006; non-parametric, paired permutation test: *p*_permutation; head/body twitches_ = 0.0032) as well as a decrease in number and duration of scratching (Wilcoxon signed-rank test, paired; *V* = 7, *p*_number of scratching bouts_ = 0.001; non-parametric, paired permutation test: *p*_permutation; scratching events_ = 0.0011; Wilcoxon signed-rank test, paired; *V* = 21, *p*_duration of scratching_ = 0.029) in Sapap3^−/−^ mice under aripiprazole treatment (Fig. 5A). However, despite a tendency, such effect was absent for the number and total duration of long grooming events (Wilcoxon signed-rank test, paired; *V* = 29, *p*_number of long groomings_ = 0.083; non-parametric, paired permutation test: *p*_permutation; long groomings_ = 0.087; Wilcoxon signed-rank test, paired; *V* = 38, *p*_duration of long groomings_ = 0.23) (Fig. 5A). In addition, we calculated effect sizes of all four RBs, which showed a lower effect on long grooming events when compared to the three other RBs (Wilcoxon effect sizes: *r*_short grooming_ = 0.73; *r*_head/body twitches_ = 0.78; *r*_scratching_ = 0.72; *r*_long grooming_ = 0.45; Supplementary Table 1). Given the potential sedative effects of aripiprazole, in addition to assessing repetitive behaviours only during awake active phases, as control parameters, we quantified the duration of sleep episodes interspersed between active behavioural episodes, which did not differ between vehicle-treated and aripiprazole-treated animals (*n* = 15 Sapap3^−/−^ mice; Wilcoxon signed-rank test, paired; *V* = 73, *p* = 0.2; non-parametric, paired permutation test: *p* = 0.45) (Supplementary Fig. 3A). We further excluded potential sedation effects by assessing trunk centre and head centre movements as a proxy for forward locomotion as well as general activity applying the DeepLabCut toolbox, which also did not differ between the vehicle and the aripiprazole condition (Wilcoxon signed-rank test, paired; *V*_trunk_ = 36, *p* = 0.2; *V*_head_ = 53, *p* = 0.5; non-parametric, paired permutation test: *p*_trunk_ = 0.2; *p*_head_ = 0.39) (Supplementary Fig. 3B, C). No correlations were observed between repetitive behaviours and activity parameters (Spearman correlation: all *p* > 0.1; all detailed information is available in Supplementary Table 1), nor did we observe any significant interaction between these activity parameters and treatment (LMM: all *p* > 0.2, all detailed information is available in Supplementary Table 1), furthermore excluding potential sedation effects in our assay. Lastly, to estimate the potentially confounding effect of potential handling and injection stress, we also tested for the interaction of treatment with injection order, but did not observe significant interactions (LMM: all *p* > 0.1; all detailed information is available in Supplementary Table 1; Supplementary Fig. 3D). Taken together, our findings suggest that specifically three out of four repetitive behaviours, which we observed as significantly present in the Sapap3^−/−^ mouse model, responded to a pharmacological treatment, which has proven success in treating tic-like movements both in Tourette syndrome in humans as well as in corresponding rodent models [56, 57, 69–72, 77]. Thus, we provide evidence that three types of RBs, namely head/body twitches, short single-phase grooming events and scratching, additionally possess predictive validity for tic-like symptoms.

## Discussion

Here, we reconsidered the current main reference mouse model of compulsive-like behaviours, the Sapap3^−/−^ mouse, in light of the cortico-striatal circuitry as a substrate for pathological RBs. Recent studies indicate that not only the associative but also the sensorimotor CSCs might be implicated in the often comorbid occurrence of compulsive-like and tic-like RBs [3, 28, 40, 78, 79]. Concretely, we performed a detailed, behavioural re-analysis of this mouse model, discovered previously undescribed types of pathologically RBs and pharmacologically challenged their nature using aripiprazole, the first-line treatment for tic-like movements [73].

The here-detected previously unreported RBs in the Sapap3^−/−^ mice consisted of single movements, which were repeatedly executed. This included sudden, rapid head/body twitches as well as hindpaw scratching, both occurring at an aberrantly high rate in Sapap3^−/−^ mice. The suddenness and rapidity of head-body twitches and their successful pharmacological treatment using aripiprazole hint straight to an interpretation of these RBs as tic-like RBs. As a marginal sedative effect, which does not impede a normal life in society, has been reported in some patients [73], we analysed and excluded potential sedation side effects of aripiprazole accounting for changes in head/body twitches and other RBs in our dataset. Replication of our pioneering findings in a larger cohort would be recommended to further substantiate our findings. The presence of both tic- and compulsive-like behaviours in the same model is in line with the clinical observation of tic-like comorbidities in both patients with Tourette Syndrome as well as with OCD [3, 49, 79, 80]. Indeed, some forms of OCD can be aetiologically related to chronic tic disorders and 10–40% of OCD cases diagnosed in childhood or during adolescence are defined as belonging to a tic-related OCD subtype [51, 52, 78, 81–86]. Patients with tic-related OCD more likely report sensory phenomena such as ‘just right’ perceptions associated with sensory stimuli or the feeling of an ‘urge’ [79, 83, 87] and may respond better to neuroleptic augmentation treatment [53, 88]. Such observation is interesting given the recent reports of increased neuronal activity of striatal projection neurons expressing dopamine D2 receptors in the Sapap3^−/−^ mouse model [18]. Within this clinical context, it is important to detect necessary subtlety in the phenotype of applied research models. Hence, the presence of both tic- and compulsive-like phenotypes in the Sapap3^−/−^ model increases its importance for studying the neurobiological basis of tic- and compulsive-like comorbidities in various disorders or these pathologically RBs.

Hindpaw scratching, nearly absent in wildtype mice, occurred at an even higher frequency than head/body twitches. The importance of this RB is furthermore elevated by the systematic and consistent improvement of skin lesions in this mouse model upon hindpaw claw dulling, suggesting at least a major and maybe even a causal role of this RB in the well-reported, flagship-like phenotype of Sapap3^−/−^ mutant mice. Further support for such interpretation comes from the observation that a large proportion of skin lesions is found on body parts, which are not touched at all during syntactic self-grooming. As the sharp nail tips grow back during the second week after nail clipping treatment, the observation of remaining skin lesions 2 weeks after hindpaw nail clipping is likely a consequence of reappearing deleterious scratching effects. However, we cannot exclude at least a contribution to skin lesion maintenance due to rodent self-grooming. Taken together, our experiments suggest that hindpaw scratching most likely provokes or is at least crucially implicated in the most visible pathological phenotype of this mouse model. Can scratching pathophysiologically be defined as a tic-like behaviour? Indeed, this RB consists of a sudden, rapidly repeated single movement and its frequency correlates with head/body twitches in both wildtype and mutant mice. Aripiprazole treatment significantly decreased both scratching frequency as well as duration. Scratching may be considered similar to pathological hair-pulling and skin-picking, which has propagated a wave of clinical discussion concerning these phenotypes in human trichotillomania patients as well as frequently comorbid OCD and/or TS patients with hair-pulling and/or skin-picking pathologies [7]. Indeed, it has been reported that patients with tic-related OCD also have higher rates of TTM [50, 80]. Interestingly, although no direct link was found between genetic *SAPAP3* variants and OCD, identified single nucleotide polymorphisms were associated with grooming disorders such as pathologic nail biting, pathologic skin-picking, and/or trichotillomania, an obsessive-compulsive related disorder [89, 90]. These genetic studies underline the potential involvement of *SAPAP3*/*Sapap3* in the generation of hair-pulling or other grooming disorders, which occur in TTM or as a comorbidity in OCD and TS patients [89]. TTM possesses clinical characteristics, which overlap with TS and OCD, e.g., the premonitory urge and temporary relief after completion of individual repetitive behaviours [91].

Having observed these previously unreported RBs in the Sapap3^−/−^ mouse model, we last revisited the syntactic self-grooming phenotype, the sole defined RB which had led to the definition of these mice as a compulsive-like model. Indeed, we confirmed the well-reported compulsive-like phenotype of an increased number of grooming bouts in these mice, however, could not replicate the increased duration of self-grooming RB, which represents the most often reported pathological parameter in Sapap3^−/−^ mice [13, 24, 54]. Most likely, the incongruence of our findings with previous reports is caused by a distinction of scratching and self-grooming behaviour, which was first performed in this study. Indeed, pooling of these two RBs has been previously mentioned [13] and pooling these two distinct repetitive behaviours in our datasets indeed results in a significant genotype-dependent difference (Mann–Whitney *U*: *W* = 65, *p* = 0.003) (Supplementary Table 1). Yet, self-grooming is a highly stereotyped linear action sequence, which follows a predictable order [62], while scratching as a single isolated action does not share these properties of linearity and predictability. Thus, pooling of two qualitatively very distinct forms of behaviour causes confounds in the behavioural phenotyping and in drawing conclusions for translational approaches.

As a last major finding of our study, we observed that self-grooming events in Sapap3^−/−^ mice were not always conformed with syntactic rodent self-grooming stricto sensu, i.e., composed of a syntactic chain of different, well-defined grooming phases [9, 62, 67]. Instead, the majority of Sapap3^−/−^ self-grooming events were of short duration and seemed to consist of a single grooming phase only, i.e., a repeatedly executed, short and single movement. Both short and long grooming events distinguish Sapap3^−/−^ from wildtype mice given their aberrant frequency, but their neurobiological nature seems to differ. Indeed, aripiprazole significantly reduced short but not long grooming events. Both the symptomatologic description of short grooming events and a decrease in their frequency upon aripiprazole treatment, i.e., face and predictive validity both suggest that short single-phase grooming events could be considered as tic-like events. On the other hand, longer grooming events, which mostly consisted of a syntactic sequence of different grooming phases, might form a category of RBs apart from the others: first, despite a tendency, this category was the only one that was not significantly reduced by acute aripiprazole administration. Secondly, effect size of the long grooming category was much smaller than the comparable effect sizes of the other three RB categories. While these results might suggest a different neurobiological nature of these two types of grooming events, conclusions of our findings on long grooming remain limited and will benefit from a follow-up, dedicated study with a much higher sample number. First, despite our negative findings, a tendency of decrease in long grooming behaviours was still detected, indicating a possible aripiprazole response also in long grooming events, maybe due to individual heterogeneity, which has previously been reported in our own work and that of others [20, 22]. Second, although in our analysis, we statistically excluded the confounding factor of handling and injection stress, we cannot entirely rule out such effects in this mouse model with marked anxiety. Altogether, our results report important evidence that self-grooming behaviour should not be considered a homogeneous behaviour and pronounce that a detailed characterisation is essential to capture its neurobiological nature. Ushering a paradigm shift in the definition of rodent self-grooming might provide deeper insights into the pathological nature of RBs. This is important for the Sapap3^−/−^ mouse as we exemplarily analysed, but might need to be considered also for other mouse models, for which aberrantly elevated self-grooming behaviour had been reported [45, 92, 93]. Thus, differentiating distinct forms of self-grooming or other behavioural phenotypes could help researchers to more adequately investigate the neurobiology of RBs [20, 22, 94].

Taken together, we observed distinct types of repetitive behaviours in the Sapap3^−/−^ mouse model, three of which can be labelled as tic-like behaviours according to face and predictive validity criteria [95]. We confirm previously reported excessive self-grooming sequences in Sapap3^−/−^ mice, but highlight the necessity to distinguish these from more sudden and simple repetitive behaviours. Indeed, we conclude that excessive number of grooming onsets rather than their duration characterises the pathological phenotype of Sapap3^−/−^ mice. This observation of exaggerated grooming onsets is in line with previous studies suggesting that Sapap3^−/−^ mice lack inhibition in executing an acquired motor sequence [16, 28]. This phenotype seems to be anchored in a diminished number of striatal parvalbumin-positive interneurons [16], which form a strong feed-forward inhibitory striatal regulatory network [96], as well as an increased striatal input of premotor cortico-striatal projections [28], a pathway which has been shown to be important for initiating behavioural sequences [97].

Altogether, the here newly reported comorbidity of different RBs in Sapap3^−/−^ mice is in line with the numerous clinical reports of comorbidity of tics and compulsions in OCD as well as TS patients [3]. These results are also in line with the current literature on disorders of repetitive behaviours, which include fundamental neuroscience studies highlighting the potential implication of sensorimotor cortico-striatal circuits. Comorbidity findings of tic- and compulsive-like behaviours in Sapap3^−/−^ further corroborate the current hypothesis of a common neurobiological basis in disorders with repetitive behaviours. Re-defining the Sapap3^−/−^ mouse as a mouse model of RBs instead of compulsive-like behaviours raises its translational value in defining the proposed common neurobiological mechanism of tic- and compulsive-like symptoms.

## Supplementary information

Supplementary Video 1

Supplementary Video 2

Supplementary Video 3

Supplementary Figure 1

Supplementary Figure 2

Supplementary Figure 3

Supplementary Figure Legends

Supplementary Table 1

## References

[1] Freeman RD, Soltanifar A, Baer S (2010). Stereotypic movement disorder: easily missed. Dev Med Child Neurol.

[2] Jiujias M, Kelley E, Hall L (2017). Restricted, repetitive behaviors in autism spectrum disorder and obsessive–compulsive disorder: a comparative review. Child Psychiatry Hum Dev.

[3] Worbe Y, Mallet L, Golmard J-L, Béhar C, Durif F, Jalenques I (2010). Repetitive behaviours in patients with Gilles de la Tourette syndrome: tics, compulsions, or both?. PLoS One.

[4] Leckman JF, Bloch MH, King RA (2009). Symptom dimensions and subtypes of obsessive-compulsive disorder: a developmental perspective. Dialogues Clin Neurosci.

[5] American Psychiatric Association, American Psychiatric Association (editors). Diagnostic and statistical manual of mental disorders: DSM-5. 5th ed. Washington, DC: American Psychiatric Association; 2013.

[6] Kircanski K, Woods DW, Chang SW, Ricketts EJ, Piacentini JC (2010). Cluster analysis of the Yale Global Tic Severity Scale (YGTSS): symptom dimensions and clinical correlates in an outpatient youth sample. J Abnorm Child Psychol.

[7] Lamothe H, Baleyte J-M, Mallet L, Pelissolo A (2020). Trichotillomania is more related to Tourette disorder than to obsessive-compulsive disorder. Braz J Psychiatry.

[8] Kalueff AV, Stewart AM, Song C, Berridge KC, Graybiel AM, Fentress JC (2016). Neurobiology of rodent self-grooming and its value for translational neuroscience. Nat Rev Neurosci.

[9] Berridge KC, Whishaw IQ (1992). Cortex, striatum and cerebellum: control of serial order in a grooming sequence. Exp Brain Res.

[10] Ting JT, Feng G (2011). Neurobiology of obsessive-compulsive disorder: insights into neural circuitry dysfunction through mouse genetics. Curr Opin Neurobiol.

[11] Milad MR, Rauch SL (2012). Obsessive-compulsive disorder: beyond segregated cortico-striatal pathways. Trends Cogn Sci.

[12] Pittenger C, Bloch MH, Williams K (2011). Glutamate abnormalities in obsessive compulsive disorder: neurobiology, pathophysiology, and treatment. Pharm Ther.

[13] Welch JM, Lu J, Rodriguiz RM, Trotta NC, Peca J, Ding J-D (2007). Cortico-striatal synaptic defects and OCD-like behaviours in Sapap3-mutant mice. Nature.

[14] Wan Y, Ade KK, Caffall Z, Ilcim Ozlu M, Eroglu C, Feng G (2014). Circuit-selective striatal synaptic dysfunction in the Sapap3 knockout mouse model of obsessive-compulsive disorder. Biol Psychiatry.

[15] Pauls DL, Abramovitch A, Rauch SL, Geller DA (2014). Obsessive-compulsive disorder: an integrative genetic and neurobiological perspective. Nat Rev Neurosci.

[16] Burguière E, Monteiro P, Feng G, Graybiel AM (2013). Optogenetic stimulation of lateral orbitofronto-striatal pathway suppresses compulsive behaviors. Science.

[17] Rauch SL, Jenike MA, Alpert NM, Baer L, Breiter HC, Savage CR (1994). Regional cerebral blood flow measured during symptom provocation in obsessive-compulsive disorder using oxygen 15-labeled carbon dioxide and positron emission tomography. Arch Gen Psychiatry.

[18] Ramírez-Armenta KI, Alatriste-León H, Verma-Rodríguez AK, Llanos-Moreno A, Ramírez-Jarquín JO, Tecuapetla F (2022). Optogenetic inhibition of indirect pathway neurons in the dorsomedial striatum reduces excessive grooming in Sapap3-knockout mice. Neuropsychopharmacology.

[19] Lousada E, Boudreau M, Cohen-Adad J, Nait Oumesmar B, Burguière E, Schreiweis C (2021). Reduced axon calibre in the associative striatum of the Sapap3 knockout mouse. Brain Sci.

[20] Manning EE, Dombrovski AY, Torregrossa MM, Ahmari SE (2019). Impaired instrumental reversal learning is associated with increased medial prefrontal cortex activity in Sapap3 knockout mouse model of compulsive behavior. Neuropsychopharmacology.

[21] van den Boom BJG, Mooij AH, Misevičiūtė I, Denys D, Willuhn I (2019). Behavioral flexibility in a mouse model for obsessive-compulsive disorder: impaired Pavlovian reversal learning in SAPAP3 mutants. Genes Brain Behav.

[22] Benzina N, N’Diaye K, Pelissolo A, Mallet L, Burguière E (2021). A cross-species assessment of behavioral flexibility in compulsive disorders. Commun Biol.

[23] Pinhal CM, van den Boom BJG, Santana-Kragelund F, Fellinger L, Bech P, Hamelink R (2018). Differential effects of deep brain stimulation of the internal capsule and the striatum on excessive grooming in Sapap3 mutant mice. Biol Psychiatry.

[24] Manning EE, Wang AY, Saikali LM, Winner AS, Ahmari SE (2021). Disruption of prepulse inhibition is associated with compulsive behavior severity and nucleus accumbens dopamine receptor changes in Sapap3 knockout mice. Sci Rep.

[25] Mallet L, Polosan M, Jaafari N, Baup N, Welter M-L, Fontaine D (2008). Subthalamic nucleus stimulation in severe obsessive-compulsive disorder. N Engl J Med.

[26] Chamberlain SR, Menzies L, Hampshire A, Suckling J, Fineberg NA, del Campo N (2008). Orbitofrontal dysfunction in patients with obsessive-compulsive disorder and their unaffected relatives. Science.

[27] Lei H, Lai J, Sun X, Xu Q, Feng G (2019). Lateral orbitofrontal dysfunction in the Sapap3 knockout mouse model of obsessive–compulsive disorder. J Psychiatry Neurosci.

[28] Corbit VL, Manning EE, Gittis AH, Ahmari SE (2019). Strengthened inputs from secondary motor cortex to striatum in a mouse model of compulsive behavior. J Neurosci.

[29] Hintiryan H, Foster NN, Bowman I, Bay M, Song MY, Gou L (2016). The mouse cortico-striatal projectome. Nat Neurosci.

[30] McGeorge AJ, Faull RL (1989). The organization of the projection from the cerebral cortex to the striatum in the rat. Neuroscience.

[31] Pan WX, Mao T, Dudman JT (2010). Inputs to the dorsal striatum of the mouse reflect the parallel circuit architecture of the forebrain. Front Neuroanat.

[32] Alexander GE, DeLong MR, Strick PL (1986). Parallel organization of functionally segregated circuits linking basal ganglia and cortex. Annu Rev Neurosci.

[33] Jin X, Costa RM (2010). Start/stop signals emerge in nigrostriatal circuits during sequence learning. Nature.

[34] Miyachi S, Hikosaka O, Miyashita K, Kárádi Z, Rand MK (1997). Differential roles of monkey striatum in learning of sequential hand movement. Exp Brain Res.

[35] Miyachi S, Hikosaka O, Lu X (2002). Differential activation of monkey striatal neurons in the early and late stages of procedural learning. Exp Brain Res.

[36] Thorn CA, Atallah H, Howe M, Graybiel AM (2010). Differential dynamics of activity changes in dorsolateral and dorsomedial striatal loops during learning. Neuron.

[37] Yin HH, Mulcare SP, Hilário MRF, Clouse E, Holloway T, Davis MI (2009). Dynamic reorganization of striatal circuits during the acquisition and consolidation of a skill. Nat Neurosci.

[38] Gillan CM, Papmeyer M, Morein-Zamir S, Sahakian BJ, Fineberg NA, Robbins TW (2011). Disruption in the balance between goal-directed behavior and habit learning in obsessive-compulsive disorder. Am J Psychiatry.

[39] Xu M, Kobets A, Du J-C, Lennington J, Li L, Banasr M (2015). Targeted ablation of cholinergic interneurons in the dorsolateral striatum produces behavioral manifestations of Tourette syndrome. Proc Natl Acad Sci USA.

[40] Hadjas LC, Schartner MM, Cand J, Creed MC, Pascoli V, Lüscher C (2020). Projection-specific deficits in synaptic transmission in adult Sapap3-knockout mice. Neuropsychopharmacology.

[41] Kalanithi PSA, Zheng W, Kataoka Y, DiFiglia M, Grantz H, Saper CB (2005). Altered parvalbumin-positive neuron distribution in basal ganglia of individuals with Tourette syndrome. Proc Natl Acad Sci USA.

[42] Kataoka Y, Kalanithi PSA, Grantz H, Schwartz ML, Saper C, Leckman JF (2010). Decreased number of parvalbumin and cholinergic interneurons in the striatum of individuals with Tourette syndrome. J Comp Neurol.

[43] Peñagarikano O, Abrahams BS, Herman EI, Winden KD, Gdalyahu A, Dong H (2011). Absence of CNTNAP2 leads to epilepsy, neuronal migration abnormalities, and core autism-related deficits. Cell.

[44] Rapanelli M, Frick LR, Pogorelov V, Ota KT, Abbasi E, Ohtsu H (2014). Dysregulated intracellular signaling in the striatum in a pathophysiologically grounded model of Tourette syndrome. Eur Neuropsychopharmacol.

[45] Shmelkov SV, Hormigo A, Jing D, Proenca CC, Bath KG, Milde T (2010). Slitrk5 deficiency impairs corticostriatal circuitry and leads to obsessive-compulsive-like behaviors in mice. Nat Med.

[46] Nordstrom EJ, Burton FH (2002). A transgenic model of comorbid Tourette’s syndrome and obsessive-compulsive disorder circuitry. Mol Psychiatry.

[47] Baldan LC, Williams KA, Gallezot J-D, Pogorelov V, Rapanelli M, Crowley M (2014). Histidine decarboxylase deficiency causes tourette syndrome: parallel findings in humans and mice. Neuron.

[48] Xu M, Li L, Ohtsu H, Pittenger C (2015). Histidine decarboxylase knockout mice, a genetic model of Tourette syndrome, show repetitive grooming after induced fear. Neurosci Lett.

[49] Leckman JF, Denys D, Simpson HB, Mataix-Cols D, Hollander E, Saxena S (2010). Obsessive-compulsive disorder: a review of the diagnostic criteria and possible subtypes and dimensional specifiers for DSM-V. Depress Anxiety.

[50] Petter T, Richter MA, Sandor P (1998). Clinical features distinguishing patients with Tourette’s syndrome and obsessive-compulsive disorder from patients with obsessive-compulsive disorder without tics. J Clin Psychiatry.

[51] Cath DC, Spinhoven P, Hoogduin CA, Landman AD, van Woerkom TC, van de Wetering BJ (2001). Repetitive behaviors in Tourette’s syndrome and OCD with and without tics: what are the differences?. Psychiatry Res.

[52] Jaisoorya TS, Reddy YCJ, Srinath S, Thennarasu K (2008). Obsessive-compulsive disorder with and without tic disorder: a comparative study from India. CNS Spectr.

[53] Bloch MH, Landeros-Weisenberger A, Kelmendi B, Coric V, Bracken MB, Leckman JF (2006). A systematic review: antipsychotic augmentation with treatment refractory obsessive-compulsive disorder. Mol Psychiatry.

[54] van den Boom BJG, Pavlidi P, Wolf CJH, Mooij AH, Willuhn I (2017). Automated classification of self-grooming in mice using open-source software. J Neurosci Methods.

[55] Ade KK, Wan Y, Hamann HC, O’Hare JK, Guo W, Quian A (2016). Increased metabotropic glutamate receptor 5 signaling underlies obsessive-compulsive disorder-like behavioral and striatal circuit abnormalities in mice. Biol Psychiatry.

[56] Rizzo F, Abaei A, Nespoli E, Fegert JM, Hengerer B, Rasche V (2017). Aripiprazole and Riluzole treatment alters behavior and neurometabolites in young ADHD rats: a longitudinal 1H-NMR spectroscopy study at 11.7T. Transl Psychiatry.

[57] Rizzo F, Nespoli E, Abaei A, Bar-Gad I, Deelchand DK, Fegert J (2018). Aripiprazole selectively reduces motor tics in a young animal model for Tourette’s syndrome and comorbid attention deficit and hyperactivity disorder. Front Neurol.

[58] Mathis A, Mamidanna P, Cury KM, Abe T, Murthy VN, Mathis MW (2018). DeepLabCut: markerless pose estimation of user-defined body parts with deep learning. Nat Neurosci.

[59] Nath T, Mathis A, Chen AC, Patel A, Bethge M, Mathis MW (2019). Using DeepLabCut for 3D markerless pose estimation across species and behaviors. Nat Protoc.

[60] He K, Zhang X, Ren S, Sun J. Deep residual learning for image recognition. IEEE Conference on Computer Vision and Pattern Recognition (CVPR), Las Vegas, NV, USA, 2016, pp. 770–8, 10.1109/CVPR.2016.90.

[61] Berridge KC (1990). Comparative fine structure of action: rules of form and sequence in the grooming patterns of six rodent species. Behaviour.

[62] Berridge KC, Fentress JC, Parr H (1987). Natural syntax rules control action sequence of rats. Behav Brain Res.

[63] Santangelo A, Bortolato M, Mosher LJ, Crescimanno G, Di Giovanni G, Cassioli E (2018). Behavioral fragmentation in the D1CT-7 mouse model of Tourette’s syndrome. CNS Neurosci Ther.

[64] Pogorelov V, Xu M, Smith HR, Buchanan GF, Pittenger C (2015). Corticostriatal interactions in the generation of tic-like behaviors after local striatal disinhibition. Exp Neurol.

[65] Orito K, Chida Y, Fujisawa C, Arkwright PD, Matsuda H (2004). A new analytical system for quantification scratching behaviour in mice. Br J Dermatol.

[66] Inagaki N, Igeta K, Shiraishi N, Kim JF, Nagao M, Nakamura N (2003). Evaluation and characterization of mouse scratching behavior by a new apparatus, MicroAct. Ski Pharm Appl Ski Physiol.

[67] Berridge KC, Aldridge JW, Houchard KR, Zhuang X (2005). Sequential super-stereotypy of an instinctive fixed action pattern in hyper-dopaminergic mutant mice: a model of obsessive compulsive disorder and Tourette’s. BMC Biol.

[68] Bortolato M, Pittenger C (2017). Modeling tics in rodents: conceptual challenges and paths forward. J Neurosci Methods.

[69] Cox JH, Seri S, Cavanna AE (2016). Safety and efficacy of aripiprazole for the treatment of pediatric Tourette syndrome and other chronic tic disorders. Pediatr Health Med Ther.

[70] Yang C-S, Huang H, Zhang L-L, Zhu C-R, Guo Q (2015). Aripiprazole for the treatment of tic disorders in children: a systematic review and meta-analysis. BMC Psychiatry.

[71] Sallee F, Kohegyi E, Zhao J, McQuade R, Cox K, Sanchez R (2017). Randomized, double-blind, placebo-controlled trial demonstrates the efficacy and safety of oral aripiprazole for the treatment of tourette’s disorder in children and adolescents. J Child Adolesc Psychopharmacol.

[72] Hartmann A, Worbe Y (2013). Pharmacological treatment of Gilles de la Tourette syndrome. Neurosci Biobehav Rev.

[73] Pringsheim T, Okun MS, Müller-Vahl K, Martino D, Jankovic J, Cavanna AE (2019). Practice guideline recommendations summary: treatment of tics in people with Tourette syndrome and chronic tic disorders. Neurology.

[74] Shapiro DA, Renock S, Arrington E, Chiodo LA, Liu L-X, Sibley DR (2003). Aripiprazole, a novel atypical antipsychotic drug with a unique and robust pharmacology. Neuropsychopharmacology.

[75] Wood M, Reavill C (2007). Aripiprazole acts as a selective dopamine D2 receptor partial agonist. Expert Opin Investig Drugs.

[76] Kirschbaum KM, Uhr M, Holthoewer D, Namendorf C, Pietrzik C, Hiemke C (2010). Pharmacokinetics of acute and sub-chronic aripiprazole in P-glycoprotein deficient mice. Neuropharmacology.

[77] Nespoli E, Rizzo F, Boeckers T, Schulze U, Hengerer B (2018). Altered dopaminergic regulation of the dorsal striatum is able to induce tic-like movements in juvenile rats. PLoS One.

[78] Diniz JB, Rosario-Campos MC, Hounie AG, Curi M, Shavitt RG, Lopes AC (2006). Chronic tics and Tourette syndrome in patients with obsessive-compulsive disorder. J Psychiatr Res.

[79] Miguel EC, do Rosário-Campos MC, Prado HS, do Valle R, Rauch SL, Coffey BJ (2000). Sensory phenomena in obsessive-compulsive disorder and Tourette’s disorder. J Clin Psychiatry.

[80] Coffey BJ, Miguel EC, Biederman J, Baer L, Rauch SL, O’Sullivan RL (1998). Tourette’s disorder with and without obsessive-compulsive disorder in adults: are they different?. J Nerv Ment Dis.

[81] Leonard HL, Lenane MC, Swedo SE, Rettew DC, Gershon ES, Rapoport JL (1992). Tics and Tourette’s disorder: a 2- to 7-year follow-up of 54 obsessive-compulsive children. Am J Psychiatry.

[82] George MS, Trimble MR, Ring HA, Sallee FR, Robertson MM (1993). Obsessions in obsessive-compulsive disorder with and without Gilles de la Tourette’s syndrome. Am J Psychiatry.

[83] Leckman JF, Grice DE, Barr LC, de Vries AL, Martin C, Cohen DJ (1994). Tic-related vs. non-tic-related obsessive compulsive disorder. Anxiety.

[84] Holzer JC, Goodman WK, McDougle CJ, Baer L, Boyarsky BK, Leckman JF (1994). Obsessive-compulsive disorder with and without a chronic tic disorder. A comparison of symptoms in 70 patients. Br J Psychiatry.

[85] Zohar AH, Pauls DL, Ratzoni G, Apter A, Dycian A, Binder M (1997). Obsessive-compulsive disorder with and without tics in an epidemiological sample of adolescents. Am J Psychiatry.

[86] Eichstedt JA, Arnold SL (2001). Childhood-onset obsessive-compulsive disorder: a tic-related subtype of OCD?. Clin Psychol Rev.

[87] Prado HS, Rosário MC, Lee J, Hounie AG, Shavitt RG, Miguel EC (2008). Sensory phenomena in obsessive-compulsive disorder and tic disorders: a review of the literature. CNS Spectr.

[88] Ipser JC, Carey P, Dhansay Y, Fakier N, Seedat S, Stein DJ (2006). Pharmacotherapy augmentation strategies in treatment-resistant anxiety disorders. Cochrane Database Syst Rev.

[89] Bienvenu OJ, Wang Y, Shugart YY, Welch JM, Grados MA, Fyer AJ (2009). Sapap3 and pathological grooming in humans: Results from the OCD collaborative genetics study. Am J Med Genet B Neuropsychiatr Genet.

[90] Boardman L, van der Merwe L, Lochner C, Kinnear CJ, Seedat S, Stein DJ (2011). Investigating SAPAP3 variants in the etiology of obsessive-compulsive disorder and trichotillomania in the South African white population. Compr Psychiatry.

[91] Novak CE, Keuthen NJ, Stewart SE, Pauls DL (2009). A twin concordance study of trichotillomania. Am J Med Genet B Neuropsychiatr Genet.

[92] Nagarajan N, Jones BW, West PJ, Marc RE, Capecchi MR (2018). Corticostriatal circuit defects in Hoxb8 mutant mice. Mol Psychiatry.

[93] Mei Y, Monteiro P, Zhou Y, Kim J-A, Gao X, Fu Z (2016). Adult restoration of Shank3 expression rescues selective autistic-like phenotypes. Nature.

[94] Schreiweis C, Burguière E (2022). Of pride and groom: the gains and limits of studying the neuroanatomy of rodent self-grooming in translational research. Neuron.

[95] Willner P (1986). Validation criteria for animal models of human mental disorders: learned helplessness as a paradigm case. Prog Neuropsychopharmacol Biol Psychiatry.

[96] Silberberg G, Bolam JP (2015). Local and afferent synaptic pathways in the striatal microcircuitry. Curr Opin Neurobiol.

[97] Rothwell PE, Hayton SJ, Sun GL, Fuccillo MV, Lim BK, Malenka RC (2015). Input- and output-specific regulation of serial order performance by corticostriatal circuits. Neuron.

