# Supplementary figures and images for: The Sapap3−/− mouse reconsidered as a comorbid model expressing a spectrum of pathological repetitive behaviours
Source: Transl Psychiatry. 2023 Jan 30;13:26. doi: 10.1038/s41398-023-02323-7 (PMC9886949; doi:10.1038/s41398-023-02323-7)

Supplementary Figure 1

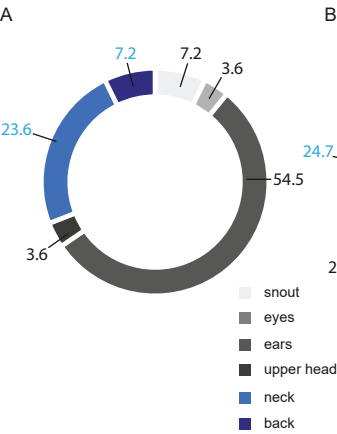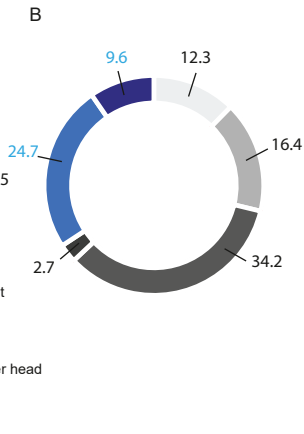

Supplement: Supplementary file 4 — Supplementary Figure 1 [file 41398_2023_2323_MOESM4_ESM.pdf]

Supplementary Figure 2

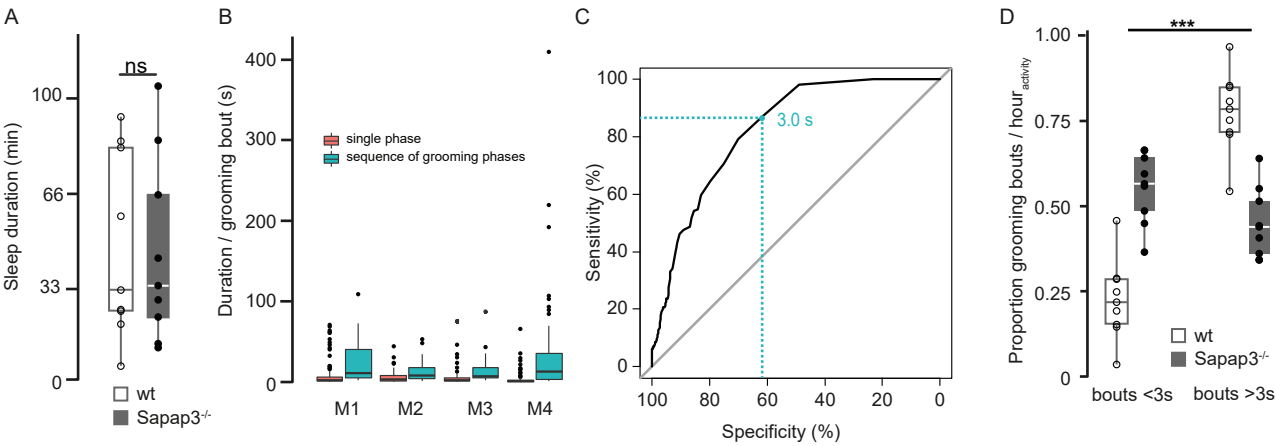

Supplement: Supplementary file 5 — Supplementary Figure 2 [file 41398_2023_2323_MOESM5_ESM.pdf]

Supplementary Figure 3

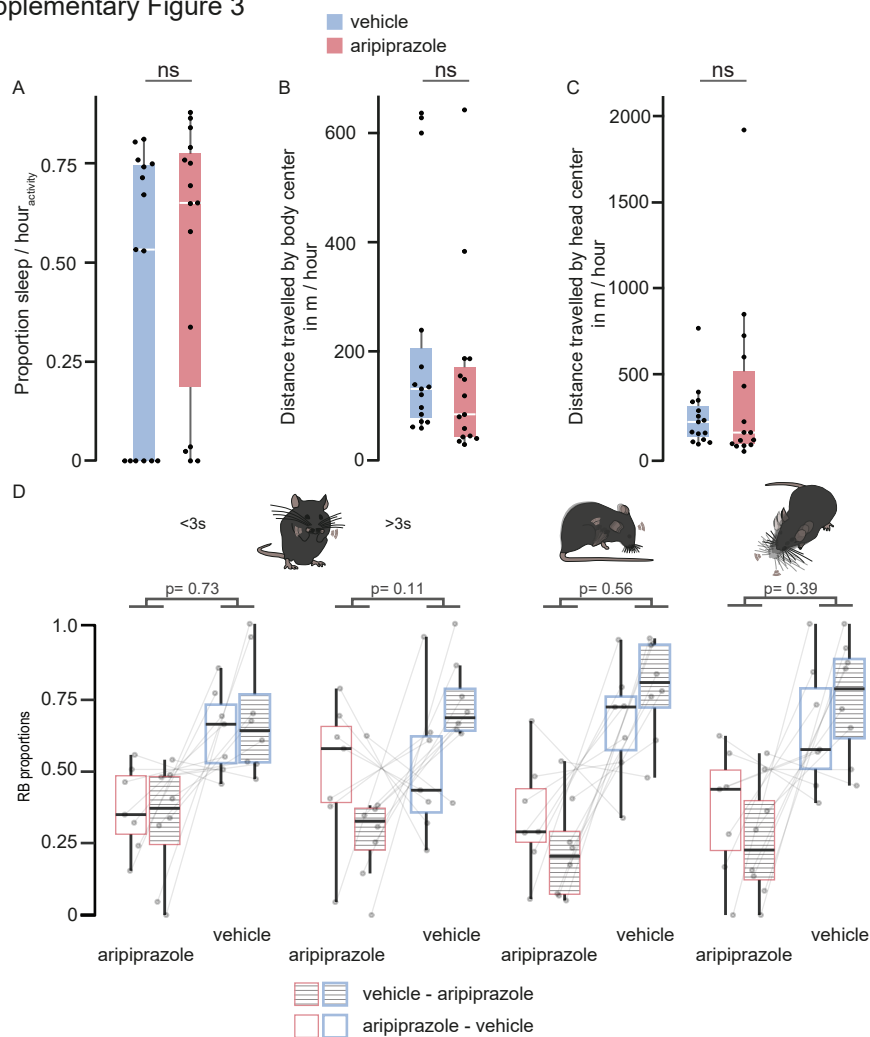

Supplement: Supplementary file 6 — Supplementary Figure 3 [file 41398_2023_2323_MOESM6_ESM.pdf]
