# Supplementary material for: The Sapap3−/− mouse reconsidered as a comorbid model expressing a spectrum of pathological repetitive behaviours
Source: Transl Psychiatry. 2023 Jan 30;13:26. doi: 10.1038/s41398-023-02323-7 (PMC9886949; doi:10.1038/s41398-023-02323-7)
Supplement: Supplementary file 7 — Supplementary Figure Legends [file 41398_2023_2323_MOESM7_ESM.docx]

**Supplementary Figure Legends**

**Supplementary Figure 1.** **Skin lesions in Sapap3-/- include body areas that are not touched during syntactic grooming**. **(A)** Distribution of most prominent skin lesions (in %) detected in a large colony pool of Sapap3-/- mice (n = 55), including snout, eye, ear, upper head, neck and back body areas. (B) In more than 80% of animals analysed in detail (n = 32), the principal lesion was accompanied by further lesions at multiple sites.

**Supplementary Figure 2.** **Distinction between short and long grooming episodes. (A)** Sleep duration was comparable in wildtype and Sapap3^-/-^ mice (Mann-Whitney U, p = ns). **(B)** Grooming events of short duration (<3 seconds) tend to consist of single-phase grooming events, while longer grooming events (>3 second) tend to consist of syntactic grooming events. **(C)** Receiver operating characteristic (ROC) curve estimation of the best cut-off separating single- and syntactic grooming bouts according to their duration at 3 seconds (true positive rate / sensitivity_3s_ = 87.2%; false positive rejection rate / Specificity_3s_ = 61.5%). (D) Sapap3-/- mice and wildtype mice significantly differ in the proportion of short, single-phase and long, syntactic grooming events (ART ANOVA, p_genotype* grooming type_ < 0.001). Box plots were illustrated as described in legend of Figure 2. *** = p < 0.001, ns = non-significant.

**Supplementary Figure 3.** **Sleep, activity and locomotor assessment exclude sedation side effects under aripiprazole. (A)** The proportion of sleep per hour of active behaviour was comparable between vehicle and aripiprazole treatment (1.5mg/kg) in Sapap3^-/-^ mice (Wilcoxon signed-rank test: p = 0.2; non-parametric, paired permutation test: p = 0.45). **(B)** Total distance travelled using DeepLabCut-based automated tracking of centre back marker as a proxy for locomotion was comparable between vehicle and aripiprazole condition (Wilcoxon signed-rank test: p = 0.2; non-parametric, paired permutation test: p = 0.2). **(C)** Comparable activity between vehicle and aripiprazole treatment as assessed through DeepLabCut-based automated tracking of head marker (Wilcoxon signed-rank test: p = 0.5; non-parametric, paired permutation test: p = 0.39). (D) No confounding influence of injection order and treatment was detected (LMM: p_grooming<3s_ = 0.73; p_grooming>3s_ = 0.11; p_scratching_ = 0.56; p_head/body twitches_ = 0.39). n = 15 Sapap3^-/-^. Box plots were followed the design described in the legend of Figure 2; the vehicle and aripiprazole conditions are colour-coded in light blue and light red, respectively. ns = non-significant.
